# The effects of collagen peptides on exercise-induced gastrointestinal stress: a randomized, controlled trial

**DOI:** 10.1007/s00394-022-03051-2

**Published:** 2022-11-12

**Authors:** Guy Taylor, Amber Leonard, Jonathan C. Y. Tang, Rachel Dunn, William D. Fraser, Nicolina Virgilio, Janne Prawitt, Emma Stevenson, Tom Clifford

**Affiliations:** 1 Institue of Population Health Sciences, Newcastle University, Newcastle, UK; 2 School of Sport, Exercise and Health Sciences, Loughborough University, Loughborough, LE11 3TU UK; 3 Norwich Medical School, University of East Anglia, Norwich University Hospital Norfolk, Norfolk, UK; 4 Rousselot BV, Ghent, Belgium

**Keywords:** Protein, Exercise, Endotoxemia, Running

## Abstract

**Purpose:**

We examined the effects of collagen peptides (CP) supplementation on exercise-induced gastrointestinal (GI) stress.

**Methods:**

In a randomized, crossover design, 20 volunteers (16 males: $\dot{V}$O_2max_, 53.4 ± 5.9 ml·kg^−1^) completed 3 trials: a non-exercise rest trial, with no supplement (REST) and then an exercise trial with CP (10 g·day^−1^) or placebo control (CON) supplements, which were consumed for 7 days prior to, and 45 min before, a 70 min run at 70–90% of $\dot{V}$O_2max_. Outcome measures included urinary lactulose and rhamnose (L/R), intestinal fatty acid binding protein (I-FABP), lipopolysaccharide (LPS), anti-LPS antibody, monocyte-chemoattractant protein-1 (MCP-1), interleukin (IL) 6 and 8, cortisol, alkaline phosphatase (ALP) (measured pre, 10 min post and 2 h post) and subjective GI symptoms.

**Results:**

There were no differences in heart rate, perceived exertion, thermal comfort, or core temperature during exercise in the CP and CON trials (all *P* > 0.05). I-FABP was higher in CP (2538 ± 1221 pg/ml) and CON (2541 ± 766 pg/ml) vs. REST 2 h post (1893 ± 1941 pg/ml) (both *P* < 0.05). LPS increased in CON vs. REST 2 h post (+ 71.8 pg/ml; *P* < 0.05). Anti-LPS antibody decreased in CON and CP vs. REST at post (both *P* < 0.05). There were no differences in MCP-1, IL-6, and IL-8 between the CP and CON trials (all *P* > 0.05), and no differences in L/R or GI symptoms between CON and CP (all *P* > 0.05).

**Conclusion:**

Collagen peptides did not modify exercise-induced changes in inflammation, GI integrity or subjective GI symptoms but LPS was higher in CON 2 h post-exercise and thus future studies may be warranted.

## Introduction

The gastrointestinal (GI) barrier is comprised of simple columnar epithelial cells densely held together by tight junction proteins [1, 2]. Together, they form an important physical barrier that separates the lumen of the intestinal tract from the circulation and tissues in the body [2]. This barrier has dual functions; on the one hand, it facilitates transportation of nutrients from the intestine to the circulation; on the other hand, it restricts the entry of potentially noxious stimuli, such as dietary antigens, endotoxins, and other bacteria [3]. If the barrier is disrupted, these toxins can leak into the circulation and initiate a cascade of inflammatory events that, if left unchecked, have been implicated in the pathogenesis of several GI disorders, including irritable bowel syndrome and celiac disease [2, 3]. More acutely, these toxins are associated with symptoms of GI stress such as abdominal pain, stomach cramps, nausea, vomiting and diarrhoea [4–6].

Intestinal barrier dysfunction can result from psychological or physiological stress [4]. With regards to the latter, it is well established that strenuous exercise disrupts GI function and increases intestinal permeability [7, 8]. The precise aetiology of exercise-induced GI dysfunction is unknown, but altered transit time and motility, visceral hypersensitivity, and splanchnic hypo-perfusion, which results in intestinal ischaemia, are likely key mechanisms [9–11]. These changes may damage the epithelial cells and tight junction proteins, leading to endotoxemia and an acute phase inflammatory response characterised by cytokine release and leukocyte recruitment [3, 9]. Such effects are thought to be responsible, at least in part, for the GI distress commonly reported by endurance athletes [12]. Indeed, GI symptoms such as stomach pain and bloating are especially common in long-distance runners, and although the prevalence and severity of GI distress varies between studies, it is estimated that 30–90% of runners suffer from GI symptoms during exercise [11, 12]. As these symptoms can negatively affect performance, and in extreme cases perturb individuals from exercising, strategies that can protect the epithelial barrier could reduce GI discomfort and enhance athletic performance.

In this regard, several dietary supplements have been tested for their effects on exercise-induced GI distress. While most studies focus on which supplements could minimise intestinal barrier dysfunction and therefore reduce the prevalence and severity of GI distress, several studies have also established which foods or supplements may aggravate GI distress and should therefore be avoided prior to exercise [13]. Most studies have focussed on supplements purported to support enterocyte integrity and reduce inflammation, such as glutamine [14–16], probiotics [17, 18], prebiotics [19], bovine colostrum [20–22] and whey protein [23]. These supplements have had limited success and in some cases commonly consumed dietary supplements such as whey protein [23] electrolytes [24] and caffeine [25] have triggered GI symptoms. Hence, recent reviews conclude that the available evidence is too limited and equivocal to recommend any specific dietary supplement for the prevention and management of GI distress during exercise [8, 13, 26]. Thus, further studies with both existing and new dietary supplements are warranted.

A novel strategy is the use of collagen peptides (CP), which contain high amounts of the amino acids glycine, hydroxyproline and proline. Intact collagen is the major component of the extracellular matrix in the body. In a recent in vitro study [27], CP attenuated intestinal barrier dysfunction in Caco-2 cell monolayers induced by tumour necrosis factor-alpha (TNF-α), a pro-inflammatory cytokine. In this model, CP fractions derived from Alaska pollock prevented the breakdown of the tight junction proteins occludin and zonulin (ZO-1) and attenuated nuclear factor kappa light chain enhancer of activated B cells (NF-κB) and extracellular regulated protein kinase ½ (ERK 1/2) signalling [27]. These findings have been corroborated by recent studies in mice, whereby CP ingestion following burn-induced GI damage maintained intestinal occludin and ZO-1 expression compared to a control [28]. A similar study also found CP to attenuate serum and intestinal inflammation [29]. Collectively, these findings suggest that CP could reinforce enterocyte integrity by modulating the expression of tight junction proteins and reducing inflammation and, as a result, could serve as a useful strategy to prevent GI damage and possibly moderate unwanted symptoms.

To date, however, no study has evaluated the effects of CP on GI integrity, permeability, or symptoms in exercising humans. Consequently, the aim of the present study was to examine whether supplementation with CP before high intensity running exercise can affect markers of inflammation, intestinal barrier dysfunction and subjective GI symptoms. As CP are increasingly being used by athletes [30, 31], we wanted to determine if CP can attenuate or exacerbate any exercise-induced GI symptoms..

## Methods

### Participants

G*power 3.1.9.2 for Microsoft Windows [32] was used to perform a priori power analysis for differences in our main primary outcome measure, I-FABP, using a two-way repeated measures ANOVA. Using the following input parameters: two tailed, α level of 0.05, beta of 0.80, and a medium to large effect size (0.65 based on Cohens *d* [33]) and estimated from a previous intervention with protein [23] it was calculated that 17 participants were required to detect statistically significant differences. In total, we recruited 20 moderately well-trained males (*n* = 16) and females (*n* = 4) (age, 29 ± 4; mass, 73.5 ± 8.5 kg; height, 1.78 ± 0.68 m; $\dot{V}$O_2max_, 53.4 ± 5.9 ml kg^−1^) who provided written informed consent to participate in this study. Participants underwent medical screening and were excluded if they had a food allergy, regularly used anti-inflammatory medications (within 2 weeks of participation), had a previous history of cardiovascular or GI complications during exercise, a diagnosed GI disease, or any other contraindication to the study procedures. The protocol received ethical approval from Newcastle University Faculty of Medical Sciences (Ethics number: 1693_1/2502/2018) and was conducted in accordance with the Declaration of Helsinki. The study was retrospectively registered on the Open Science Framework titled: Collagen supplementation and exercise-induced gastrointestinal distress (https://osf.io/wug46/).

### Experimental design

**Fig. 1:**
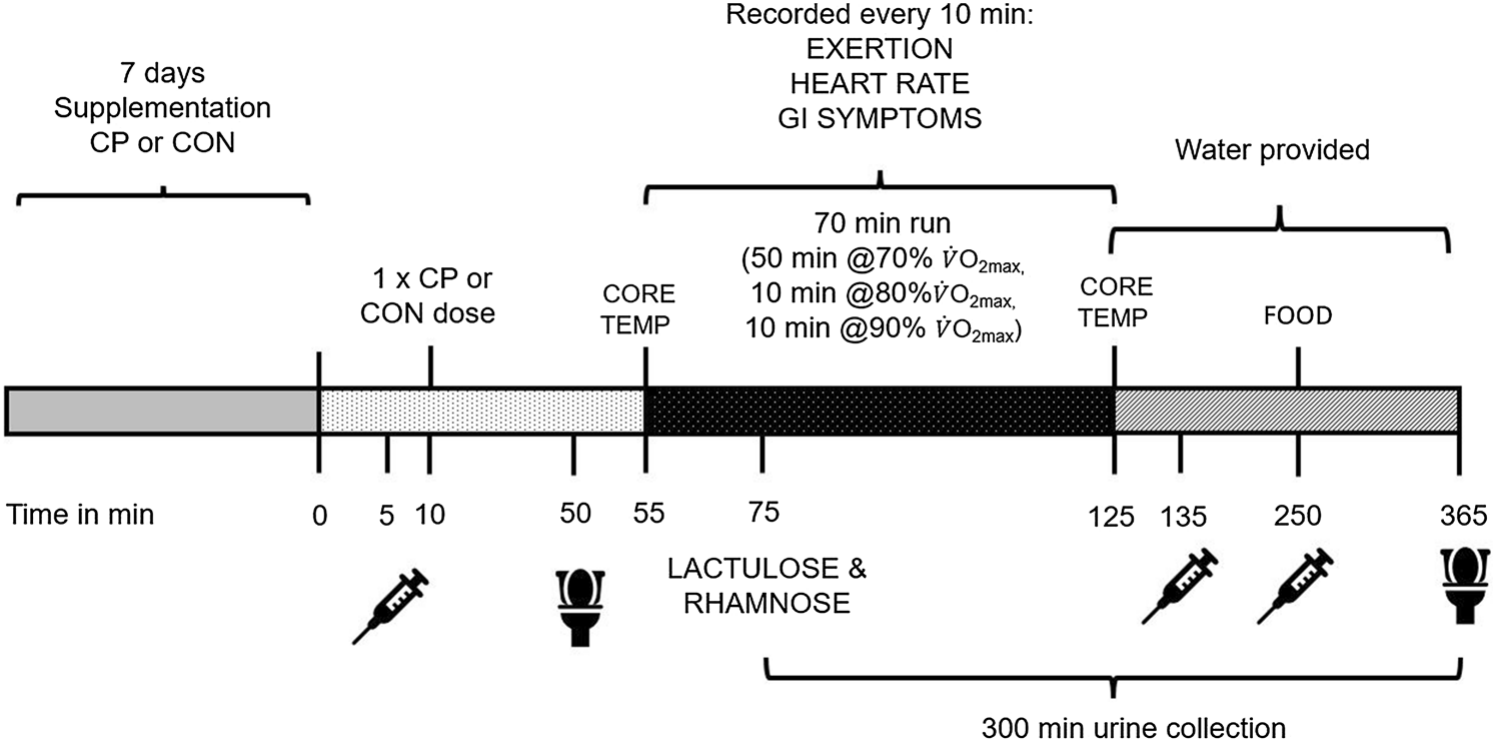
Schematic outline of procedures for the exercise trials: collagen peptides (CP) and control (CON). CP and CON trials were performed in a crossover fashion; the second trial was repeated after a washout phase of 14 days. Time is presented in minutes. The CP and CON trials (7-day supplementation) were performed ≥ 7 days after a REST, no supplement, and no exercise trial. GI, gastrointestinal; core temp, core temperature recorded; Food, food provided; exertion, rate of perceived exertion

This study employed a randomized, double blind, placebo controlled, crossover design with two experimental treatment arms. Following preliminary testing, participants attended the lab for 3 main trials. On the first trial, non-exercise, no supplement, resting data was collected (REST). On the following 2 trials, the procedures were identical to the REST trial except participants ran on a treadmill for 70 min to induce GI distress and consumed supplements. For these two exercise trials participants were randomized to receive either 10 g·day^−1^ of CP or a flavour matched placebo control (CON) for 7 days prior to the trial, and on the trial day 45 min before exercise. On the REST trial, the supplement was substituted for water. A schematic outline of the study procedures for the two supplement, exercise conditions, is displayed in Fig. 1. The order for these supplements in the crossover design was randomly generated by online software (Graphpad Prism, CA, US). Participants recorded their dietary intake in the 24 h before the REST trial and replicated this intake in the 2 subsequent exercise trials. Participants avoided strenuous exercise and alcohol intake 48 h prior to all testing sessions and consuming any other dietary supplements during the testing period.

### Preliminary testing

After collecting measures of height and body mass, participants completed a maximal aerobic capacity test ($\dot{V}$O_2max_) on a motorised treadmill. To assess $\dot{V}$O_2max_, participants ran at a self-selected speed (Km∙h^−1^) and then the treadmill grade was increased by 1% every minute until volitional fatigue or when heart rate (HR) reached 10 beats·min^−1^ of age predicted max, or respiratory exchange ratio was > 1.1. Expired gases were collected throughout with a metabolic cart (Vyntus CPX, Norwood, UK).

### Main trials

Approximately ~ 10 h before each main trial participants ingested a telemetric pill (Core Body Temperature Sensor, HQ Inc, Palmetto, FL) to assess core temperature, as described previously [34]. Participants arrived at the lab following an overnight fast and consumed 500 ml of water within 30 min of their visit. All testing started between 07:00–09:00 and was conducted at an ambient temperature of 22.1 ± 1.0. During the REST trials, participants were seated and rested. During the two exercise trials, participants ran for 70 min (50 min at 70%, 10 min at 80% and 10 min at 90 of their $\dot{V}$O_2max_; fractions calculated using the formula from the American College of Sports Medicine [35]). We adapted this protocol from previous studies that showed running for this duration and intensity is necessary to increase biological markers of GI disturbance [7, 36, 37]. We also restricted fluid [37] and any other nutritional intake during exercise to exacerbate these symptoms [26].

During the 70 min exercise and REST trials, participants completed a survey to assess GI symptoms and thermal sensation [38]. The GI survey has been used in several previous studies [6, 23] and was recently validated as a reliable measure of GI symptoms during exercise [38]. Thermal comfort was assessed with a 1–8 likert scale anchored by unbearably cold (0) and unbearably hot (8) [39]. A venous blood sample was taken before they consumed 100 ml of water (REST trial), or their supplement (CP or CON trials). Participants were then fitted with a HR monitor (Polar, Kempele, Finland) and rested in the laboratory.

At 45 min post-ingestion, they either sat quietly (REST) or ran for 70 min, as described above. Twenty min into the exercise or REST trials, they consumed a 50 ml solution containing 5 g of lactulose (Sadnoz Ltd, Camberly, UK) and 2 g of rhamnose (Sigma-Aldrich, St. Louis, US). We provided the sugar solution twenty min into the exercise as this is when intestinal permeability is likely to increase [7, 16]. Rate of perceived exertion (RPE) and HR (exercise trials only) and GI symptoms and thermal comfort were recorded every 10 min during the 70 min exercise or REST period.

Immediately following the 70 min exercise or REST trials, core temperature was recorded, and nude body mass (wearing underwear only) was measured with medical weighing scales (Seca 875, Seca, Birmingham, UK). Blood samples were collected 10 and 120 min later. Water was allowed ad libitum after the 70 min period during the REST trial; for the exercise trials, participants consumed 125% of body mass lost (in water) following the run [36]. After the 120 min post blood sample, participants were provided non-sugar containing food (Roast Sliced Chicken Breast, Tesco PLC, Welwyn Garden City, Herts). Food was allowed ad libitum on the first trial and the amount was recorded and replicated in the two subsequent trials. The participants remained in the lab for 5 h after consuming the sugar solution; all urine produced in this time was collected for later analysis. The time point PRE represents pre-exercise/rest, POST is 10 min post-exercise/rest, and 2 H POST is 2-h after exercise or rest. The two intervention trials were separated by ≥ 14 days to allow for sufficient washout.

### Blood and urine collection

Blood samples were obtained from a branch of the basilica vein at the antecubital fossa using the venipuncture technique. At all 3 time points (pre, 10 min post, 2-h post), blood was drawn into a 10 ml vacutainer for serum and a 10 ml vacutainer lined with EDTA. Samples were centrifuged at 4000 rpm for 10 min to separate the supernatant, which was subsequently stored in aliquots at −80° and then used for analysis of intestinal fatty acid binding protein (I-FABP) and lipopolysaccharide (LPS) concentrations and cytokine analysis (IL-1β, IL-6, IL-8 and monocyte-chemoattractant protein-1 (MCP-1)). On the exercise trials, whole blood was taken to assess haemoglobin and haematocrit; this data was used to adjust blood markers for plasma volume changes using the equations from Dill and Costill [40]. Urine was kept refrigerated during the trials and subsequently aliquoted into micro tubes and frozen at −80°.

### Blood and urine analysis

Haemoglobin and haematocrit were measured with a Hemo Control analyser (Hemo Control, EKF diagnostic, Cardiff, UK). The coefficient of variation (CV) for this analysis was < 4%. Cortisol is a glucocorticoid hormone that increases in response to a stress, such as exercise and alkaline phosphatase (ALP) is suggested to play a role in detoxifying LPS [45]. Cortisol and ALP were measured on a Roche Cobas 8000 automated chemistry analyser (Roche Diagnostics, Indianapolis, US); performance data from the analyser show that CVs for this analysis is < 6%.

### Serum I-FABP and LPS

I-FABP is a surrogate marker of enterocyte damage and was analysed in serum with a Quantikine human ELISA kit (R&D Systems, Inc. Minneapolis, MS, USA), with inter-assay CV ≤ 10% across the assay working range of 3.6–1000 pg/mL. Systemic LPS concentrations are used as a surrogate marker of GI distress, with increases suggesting greater disruptions in GI integrity. Serum LPS was measured by CusaBio ELISA kit (CusaBio Technology LLC, Houston, TX, USA), with inter-assay CV of ≤ 12% across the measurement range of 6.3–400 pg/mL. Anti-LPS antibodies were measured with a commercially available ELISA kit (HK504, EndoCAb® IgM, Hycult Biotech, Uden, Netherlands); CV for this analysis was ≤ 7.2%.

### Urine lactulose and rhamnose

Urinary excretion of orally consumed lactulose and rhamnose is a well-established measure of intestinal permeability [41, 42]. Lactulose is thought to enter the circulation paracellularly and rhamnose transcellularly. An increase in the larger sugar, lactulose, relative to the smaller sugar, rhamnose, indicates increased intestinal permeability. This analysis was completed by a liquid chromatography tandem mass spectrometry (LC–MS/MS) method. Sample analysis was performed using a Waters Acquity I-class UPLC system coupled to the Xevo TQ-XS tandem mass spectrometer (Waters Corp., Milford, MA, USA) operated in negative electrospray mode. Chromatographic separation was achieved using a Raptor biphenyl 2.7 µm, 100 × 3.0 mm (Bellefonte, PA, USA) column heated at 50˚C. Mobile phases used were (A) water and (B) methanol in 2 mM ammonium formate, pumped at the flow rate of 0.4 mL/min in 80:20% (A:B), gradually increased to 100% (B) then returned to the starting gradient at 3 min. Before analysis, all samples underwent isotopic dilution protein precipitation procedure, whereby 10 µL of calibration standard/QC materials/study sample was added to 250 µL of ^13^C_6_-rhamnose (Omicron Biochemicals, Inc. South Bend, IN, USA) and ^13^C_12_-Lactulose (Santa Cruz Biotechnology, Inc. Dallas, TX, USA) in 80:20 acetonitrile/water internal standard mixture (1 µmol/L). After a vigour vortex followed by centrifugation (5 min at 10,000×*g*), 10 µL of the supernatant was injected in the LC–MS/MS. Argon gas was applied to the collision cell during the Collision Induced Dissociation (CID) process. Detection was based on the mass to charge (m/z) precursor to product ion transitions specific to each compound: rhamnose (163 > 103), ^13^C_6_-rhamnose (169 > 107), lactulose (341 > 161) and ^13^C_12_-lactulose (353 > 167). MassLynx version 4.2 and QuanLynx software (Waters Corp., Milford, MA, USA) were used for system control, data acquisition, baseline integration and peak quantification. The lactulose assay performed with an inter-assay CV of ≤ 5.5% across the measurement range of 0.125—50 µmol/L. Rhamnose with an inter-assay CV of ≤ 1.9% across the measurement range of 0.125–50 µmol/L.

Urine lactulose and rhamnose results obtained from LC–MS/MS analysis were adjusted for variations in renal function by dividing by urine creatinine. Urine creatinine was analysed using Roche 2nd generation kinetic colorimetric assay based on the Jaffé method performed on the COBAS® C501 analyser (Roche, Burgess Hill, UK). The inter-assay CV ranged from 1.3–2.1% and intra-assay ranged between 1.6–4% across the assay working range. External quality control (UK NEQAS) return showed an inter-laboratory CV of 2.2%. Values used for analysis were calculated by multiplying the amount of lactulose and rhamnose recovered in urine by the volume of urine produced, and then dividing this by the amount (g) consumed. Data are presented as mg/L.

### Cytokines

Numerous studies have shown that cytokines increase in response to exercise [43, 44]. IL-1β, IL-6, IL-8 and MCP-1 were analysed in EDTA treated plasma using BD™ Cytometric Bead Array (CBA) immunoassay Flex Sets according to the manufacturing instructions and as previously described [45]. Panel one consisted of enhanced sensitivity IL-1β and IL-6 (Enhanced Sensitivity Human Soluble Protein CBA Flex Set, BD Biosciences; CA, USA). Panel two consisted of IL-8 and MCP-1 (Human Soluble Protein CBA, BD Biosciences; CA, USA). All samples were acquired using the BD Accuri™ C6 Flow Cytometer (BD Biosciences; CA, USA). Data collected using the flow cytometer was analysed using FCAP Array™ Software Version 3.0 (BD Biosciences; CA, USA) for median fluorescence intensity (MFI) which was used in a 5-parameter logistic (5PL) fitted standard curve to determine cytokine concentrations.

### Dietary supplements

The CP and CON supplements were provided by Rousselot BV (Ghent, Belgium). Each serving of CP was 100 ml and contained 10 g of collagen peptides derived from bovine hide. We chose a 10 g dose because testing by the manufacturer has shown this dose to be safe and not result in adverse effects. The CON contained no active ingredients but had the same volume, and similar appearance and taste as the CP. Each bottle was labelled by the manufacturers and the supplement code was blinded from the investigators and participants until study completion. Participants consumed their respective supplements immediately upon waking for the 7 days prior to the CP and CON trials and then once more on the morning of the trial (8 days in total). For the REST trials, participants consumed 100 ml of water at the same time-points.

### Data analysis

Statistical significance was set at P < 0.05 and data were analysed using IBM SPSS Statistics 24 for Windows (Surrey, UK) and displayed as mean ± SD. Normality was assessed by inspecting histograms, skewness and kurtosis and the Shapiro–Wilk test (P < 0.05 considered not normally distributed). A repeated measures analysis of variance (ANOVA) was used to assess time (PRE, POST, 2 H POST), condition (REST, CP, CON) and time*condition interaction effects for core temperature, plasma volume, body mass, HR, RPE, thermal comfort and all blood and urine biomarkers. Aside from plasma volume and body mass, all other outcomes were not normally distributed and transformed prior to analysis to reduce skewness. A one-way ANOVA was used to assess condition (REST, CP, CON) effects for 5 h fluid intake. Gastrointestinal symptoms (GIS) data was not normally distributed and analysed using Friedman’s non-parametric tests. In the event of significant effects, post-hoc paired *t* tests or Wilcoxon signed rank tests with Bonferroni corrections were performed to locate the differences. Where relevant, if sphericity was violated the Greenhouse Geisser correction was used. Where possible, unbiased estimates of effect size (Hedges *g*) for raw mean ± SD are presented alongside results of post-hoc analysis of the main outcomes. In instances where several effect sizes are presented together, we have used the ≥ symbol to demonstrate the smallest such effect. Hedges *g* values of 0.20, 0.50 and 0.80 were considered small, medium, and large, respectively [46].

## Results

All participants completed the testing and unless otherwise stated, data is reported for all 20 volunteers.

### Body mass, core temperature, fluid intake and urine output

Changes in body mass, core temperature, fluid intakes and urine output across the three trials are presented in Table 1. As expected, core temperature increased post-exercise (time effect; *P* < 0.001; condition effect; *P* = 0.009; interaction; *P* < 0.001). Core temperature increased in CP vs. REST (*P* < 0.001) and CON vs. REST (*P* < 0.001) but there were no differences between CP and CON (*P* = 1.000).

**Table 1:** Changes in body mass, core temperature and fluid intakes and urine collection volumes across the three trials

	CP	CON	REST
Body mass loss (kg)			
Pre	73.3 ± 8.4	73.5 ± 8.6	73.3 ± 8.6
Post	71.9 ± 8.2	72.1 ± 8.3	72.9 ± 8.6
Change (%)	−1.9 ± 0.4^b^	−1.9 ± 0.4^b^	−0.6 ± 0.4^a^
Core temperature (°C)			
Pre	36.9 ± 0.7	36.8 ± 0.8	37.0 ± 0.9
Post	38.7 ± 0.6	38.6 ± 0.9	37.0 ± 1.0
Change (%)	4.6 ± 1.5^b^	4.5 ± 1.5^b^	0.1 ± 0.5^a^
5 h Fluid intake (ml)	1934 ± 488	1903 ± 462	1200 ± 394^a^
5 h Urine collection (ml)	750 ± 407	833 ± 392	1225 ± 409^a^

*CP* collagen peptides trial, *CON* control trial; *REST* no exercise rest trial

^a^Different to CP and CON trials (*P* > 0.05)

^b^Different to Pre

Pre-post-exercise changes in body mass were evident (time effect; *P* < 0.001). An interaction effect (*P* < 0.001) showed that body mass losses were greater in CP vs. REST (*P* = 0.009) and CON vs. REST (*P* = 0.002) but no differences were found for CP vs. CON (*P* = 1.000) (Table 1).

There was a condition effect (*P* < 0.001) for fluid intakes and urine output in the 5 h after consuming lactulose and rhamnose; intake and output in the CP and CON trials were greater than REST (*P* < 0.001) but not different between CP and CON for intake (*P* = 1.000) or urine output (*P* = 0.760).

### Plasma volume

Plasma volume decreased in both conditions post-run (CP: −5.3 ± 8.0% vs. CON: −6.6 ± 5.5%; time effect; *P* = 0.005) but was no different to pre-exercise levels by 2-h post-exercise (CP: 1.5 ± 4.5% vs. CON: 0.4 ± 8.6%). No condition (*P* = 0.307) or interaction effects were observed (*P* = 0.810).

### HR, RPE and thermal comfort

**Fig. 2:**
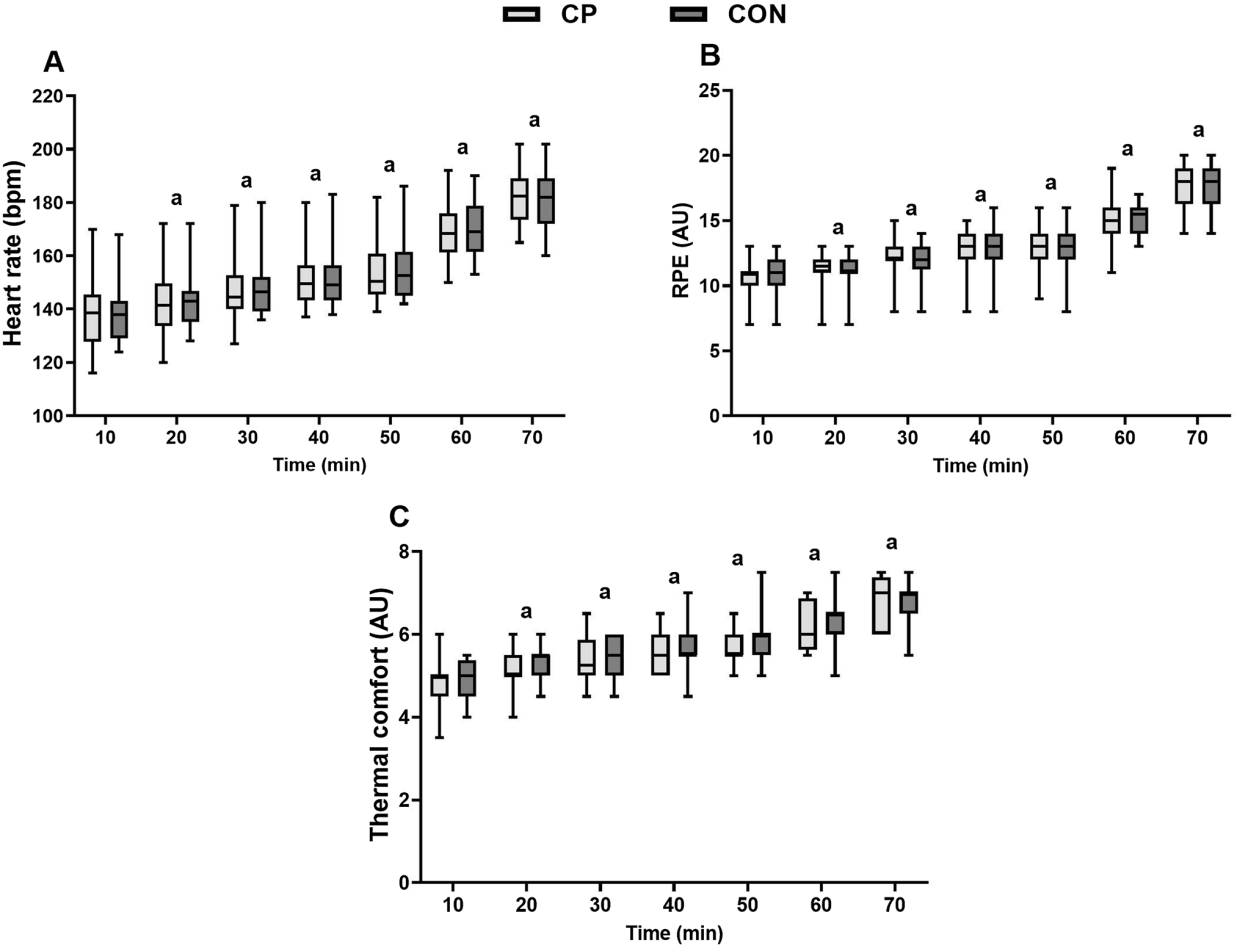
Changes in heart rate (HR) (**A**), rate of perceived exertion (RPE) (**B**) and thermal comfort (**C**) every 10 min during the 70 min of running in the collagen peptides (CP) and control (CON) trials. AU = arbitrary units; a = time effect, different to 10 min (*P* < 0.05). Data was transformed for analysis

HR increased during exercise (time effect; *P* < 0.001) but there were no differences in the CP and CON trials (condition effect; *P* = 0.818; interaction effect; *P* = 0.875; Fig. 2A). RPE also increased during exercise (time effect; *P* < 0.001) but did not differ in the CP and CON trials (condition; *P* = 0.320; interaction; *P* = 0.344; Fig. 2B). Thermal comfort deteriorated during exercise (time effect; *P* < 0.001) but there was no condition (*P* = 0.486) or interaction effects (*P* = 0.857). (Fig. 2C).

### Lactulose and rhamnose permeability test

**Fig. 3:**
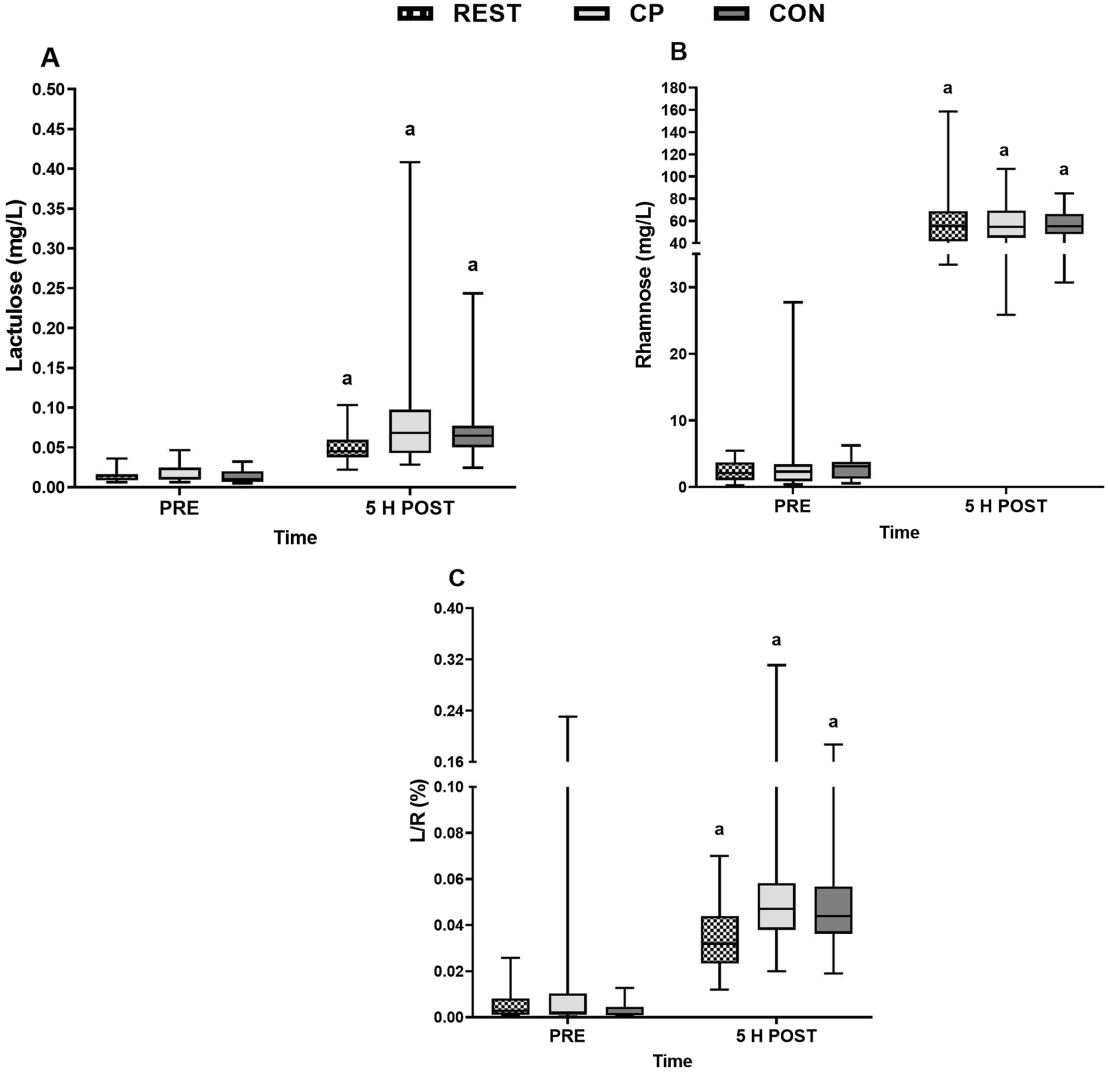
Changes in lactulose (**A**), rhamnose (**B**), and the lactulose rhamnose ratio (L/R) (**C**), before (PRE) and 5 h post lactulose and rhamnose intake (5 H POST) during a non-exercise rest trial (REST) or 70 min of running after ingesting collagen peptides (CP) or a control (CON). a = time effect, different to PRE (*P* < 0.05). Data was transformed for analysis

Lactulose and rhamnose showed time effects (*P* < 0.001), increasing in all 3 conditions (*P* ≤ 0.001; Fig. 3A, B). There was an interaction effect for lactulose (*P* = 0.046) but after post-hoc follow up, no between condition differences were observed for REST vs. CON and REST vs. CP (*P* = 0.105 and *P* = 0.116, respectively). There were medium effect sizes for an increase in CP and CON vs. REST, however (*g* = 0.56 and 0.64, respectively) suggesting the exercise likely had a meaningful impact on GI permeability. Rhamnose showed no interaction or condition effects (both *P* > 0.05). The lactulose rhamnose ratio (L/R) increased in all conditions (*P* < 0.001) but there was no condition (*P* = 0.266) or interaction effects (*P* = 0.148) (Fig. 3C).

### I-FABP

**Fig. 4:**
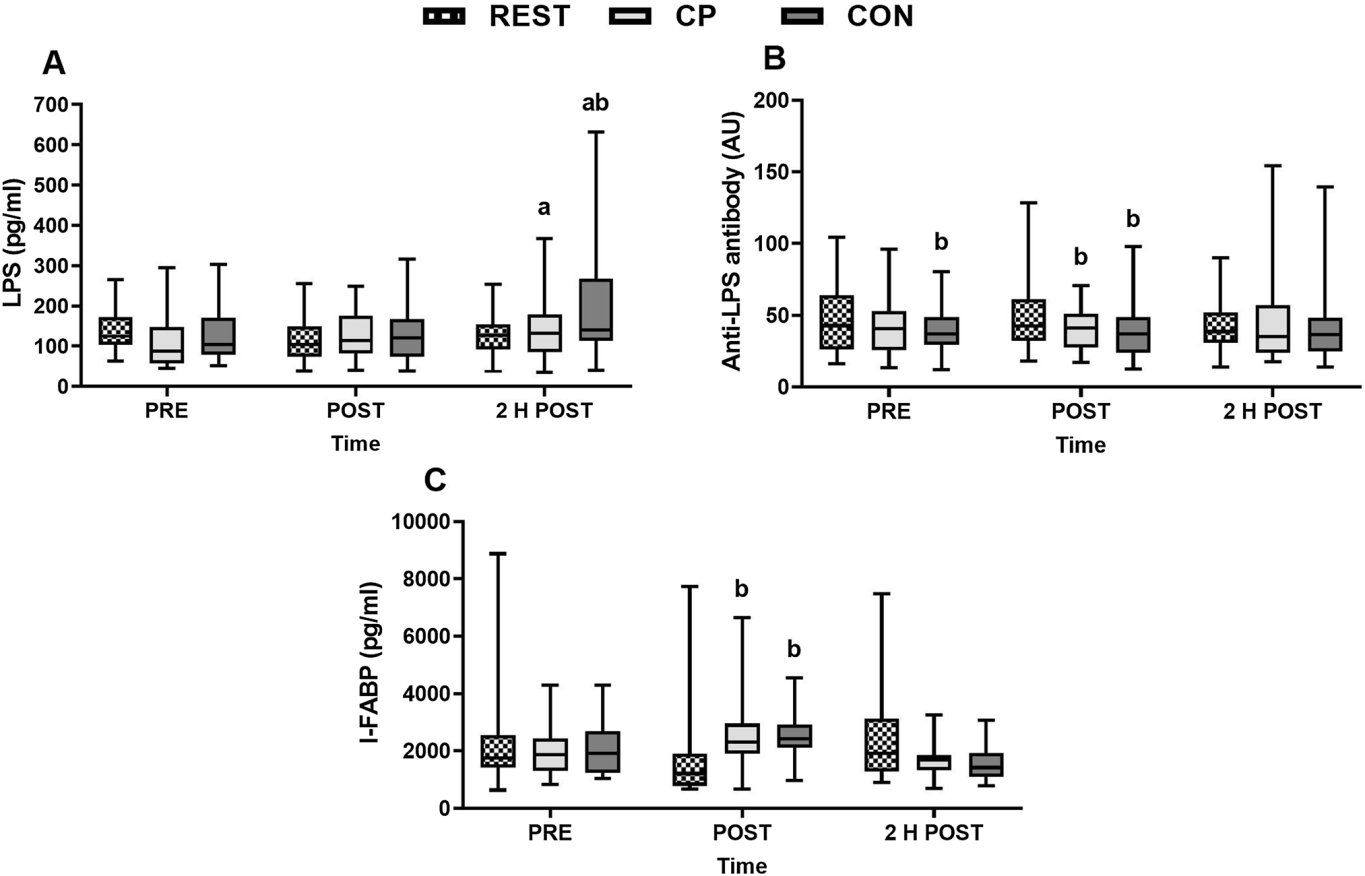
Changes in lipopolysaccharide (LPS) (**A**), anti-lipopolysaccharide antibody (anti-LPS antibody) (**B**), and intestinal fatty acid binding protein (I-FABP) (**C**) before (PRE), 10 min post (POST) and 2 h post (2 H POST) 70 min of seated rest (REST) or 70 min of running after ingesting collagen peptides (CP) or a control (CON). AU = arbitrary units; a = time effect, different to PRE (*P* < 0.05); b = interaction effect, different to REST (*P* < 0.05). Data was transformed for analysis. I-FABP data presented for *n* = 19 participants

One participant’s data was excluded due to technical difficulties during the analysis. I-FABP did not show overall time (*P* = 0.124) or condition (*P* = 0.493) effects, but an interaction was found (*P* ≤ 0.0001) with post-hoc analysis showing that at POST I-FABP levels were greater in CON vs. REST (*P* = 0.002; *g* = 0.42) and CP vs. REST (*P* = 0.022; *g* = 0.38) but not CON vs. CP (*P* = 1.000; *g* = 0.000) (Fig. 4C), suggesting no effect of the CP supplement on intestinal integrity.

### Lipopolysaccharide and anti-lipopolysaccharide antibody

LPS showed time (*P* = 0.001), condition (*P* = 0.045) and interaction effects (*P* = 0.001; Fig. 4A). LPS was higher 2 H POST than PRE in CON and CP trials (both *P* < 0.05; *g* ≥ 0.44). LPS was also higher in CON 2 H POST compared to REST (*P* = 0.019;* g* = 0.65). The difference between CP and CON at 2 H POST was not statistically significant (*P* = 0.063; *g* = 0.48). When systemic LPS increases, anti-LPS antibodies are expected to decrease, due to endotoxins consuming a large proportion of the antibodies. Anti-LPS antibody showed no time (*P* = 0.883) or interaction (*P* = 0.150) effects, but a condition effect was found (*P* = 0.005). Post-hoc tests showed that anti-LPS antibody was lower in CON vs. REST at PRE (*P* = 0.011; *g* = 0.37) and POST (*P* = 0.001; *g* = 0.43) and CP vs. REST at POST (*P* = 0.023; *g* = 0.40) (Fig. 4B).

### GI symptoms

Table 2 displays the GI symptoms reported for each condition. The data represents an aggregate of scores recorded every 10 min during the 70 min exercise or rest period. Post-hoc tests showed that total upper GI symptoms, belching and total for symptoms labelled as other (nausea, dizziness, and stitch) were all higher during exercise compared to the REST trial (all *P* < 0.05). Although a main effect was present, after adjusting for multiple comparisons, there were no significant condition differences for nausea, intestinal pain, dizziness, and abdominal stitch. Overall, there were no differences in GI symptoms between CON and CP.

**Table 2:** Subjective gastrointestinal symptoms

	CP		CON		REST		
	Symptom incidence^a^ (%)	Ratings^b^(AU)	Symptom incidence^a^(%)	Ratings^b^(AU)	Symptom incidence^a^ (%)	Ratings^b^(AU)	*P* value
Total symptoms	80	649 (0–125)	80	554 (0–116)	55	193 (0–44)	0.094
Upper Gl symptoms	70	243* (0–56)	80	215* (0–46)	40	81 (0–18)	0.001
Belching	60	71* (0–16)	60	49* (0–9)	20	13 (0–6)	0.001
Heartburn	35	27 (0–6)	20	25 (0–8)	10	9 (0–7)	0.507
Bloating	45	51 (0–17)	40	59 (0–20)	25	27 (0–14)	0.406
Stomach pain	40	42 (0–13)	45	49 (0–18)	25	18 (0–7)	0.169
Urge to regurgitate	25	31 (0–14)	25	23 (0–10)	10	7 (0–5)	0.163
Regurgitation	15	18 (0–11)	10	8 (0–6)	10	5 (0–3)	0.465
Projectile vomiting	5	3 (0–3)	5	2 (0–2)	5	2 (0–2)	0.867
Lower GI symptoms	60	269 (0–56)	70	187 (0–62)	50	112 (0–30)	0.121
Flatulence	55	82 (0–17)	65	46 (0–13)	35	26(0–8)	0.124
Lower abdominal bloating	30	53 (0–16)	30	41 (0–14)	30	22 (0–10)	0.784
Urge to defecate	55	65 (0–17)	40	39 (0–15)	30	64 (0–25)	0.196
Intestinal pain	45	69* (0–28)	40	61* (0–33)	0	0 (0–0)	0.042
Abnormal defecation^c^	0	0 (0–0)	0	0 (0–0)	0	0 (0–0)	1.000
Other GI symptoms	55	137* (0–32)	65	152* (0–32)	0	0 (0–0)	0.001
Nausea	35	59 (0–12)	30	36 (0–10)	0	0 (0–0)	0.015
Dizziness	20	31 (0–14)	35	42 (0–9)	0	0 (0–0)	0.012
Abdominal stitch	35	47 (0–14)	45	74 (0–19)	0	0 (0–0)	0.004

*Different to REST with post-hoc Wilcoxon signed rank test (*P* < 0.05)

^a^Percentage of participants reporting ≥ 1/10 for symptoms at any time point during the run

^b^Sum of each participant’s aggregate scores reported during the 70 min exercise or rest trial with the lowest to highest individual range in parenthesis

^c^Abnormal defecation including loose watery stools, diarrhoea, and blood in stools. AU, arbitrary units. *n* = 20

### Cortisol and alkaline phosphatase

Serum cortisol showed significant time (*P* < 0.001), condition (*P* = 0.016) and interaction effects (*P* < 0.001). Post-hoc analysis showed that cortisol decreased compared to PRE both POST and 2 H POST in the REST trial (*P* ≤ 0.001; *g* ≥ 0.78; Table 3) and 2 H POST in the CON and CP trials (*P* < 0.0001; *g* ≥ 2.17). At POST cortisol was higher in CP vs REST (*P* = 0.008; *g* = 2.12) and CON vs. REST (*P* = 0.003;* g* = 2.46). There were no differences in CP and CON at any time point (all *P* > 0.05). ALP showed no time (*P* = 0.098), interaction (*P* = 0.150) or condition effects (*P* = 0.767).

**Table 3:** Changes in cortisol, alkaline phosphatase (ALP), monocyte-chemoattractant protein-1 (MCP-1), interleukin-6 (IL-6), interleukin-8 (IL-8) in the three trials

	CP	CON	REST
Cortisol (nmol/L)			
Pre	413.2 ± 127.0	392.4 ± 105.1	403.0 ± 157.7
Post	446.3 ± 135.8^ab^	428.3 ± 106.0^ab^	295.5 ± 97.8a
2 h post	287.1 ± 103.0^a^	261.9 ± 63.7^a^	261.6 ± 59.5^a^
ALP (IU/L)			
Pre	66.7 ± 17.3	65.5 ± 16.4	64.1 ± 18.2
Post	66.0 ± 17.6	65.4 ± 17.3	66.8 ± 18.4
2 h post	67.5 ± 18.3	67.0 ± 16.7	67.4 ± 18.4
MCP-1 (pg/ml)			
Pre	17.0 ± 11.4	14.6 ± 9.0	18.1 ± 12.6
Post	24.6 ± 13.7^ab^	25.4 ± 15.1^ab^	11.4 ± 5.8^a^
2 h post	19.1 ± 12.3^b^	19.1 ± 10.3^ab^	10.7 ± 5.5^a^
IL-6 (pg/ml)			
Pre	3.6 ± 6.4	4.0 ± 6.7	4.6 ± 8.2
Post	6.2 ± 6.5^ab^	7.6 ± 8.6^ab^	3.9 ± 6.5
2 h post	4.6 ± 5.5^ab^	5.1 ± 4.7^ab^	3.7 ± 6.7
IL-8 (pg/ml)^#^			
Pre	4.4 ± 2.9	4.3 ± 2.1	4.2 ± 2.1
Post	5.1 ± 2.5	5.0 ± 2.3^a^	4.0 ± 2.2
2 h post	4.9 ± 2.6	5.5 ± 3.1^a^	4.1 ± 2.2

*CP* collagen peptides trial, *CON* control trial, *REST* no exercise rest trial.

^#^*n* = 17 Due to analytical difficulties

^a^Time effects vs. pre: *P* < 0.05

^b^Interaction effect vs. rest: *P* < 0.05

### Cytokines

As > 80% of samples were below the limit of detection for IL-1β, this data was not analysed. In the present study, IL-6 showed time, condition, and interaction effects (all P < 0.001). IL-6 increased POST and 2 H POST in the CON and CP trials vs. PRE (all *P* < 0.001; *g* ≥ 0.15). IL-6 was higher in CP vs. REST at POST (*P* < 0.001; *g* = 0.32) and 2 H POST (*P* = 0.026; *g* = 0.12); CON was higher vs. REST at POST (*P* < 0.001; *g* = 0.46) and 2 H POST (*P* = 0.001; *g* = 0.21). There were no differences in CP and CON at any time point (all *P* > 0.05).

IL-8, a pro-inflammatory cytokine, showed time (*P* = 0.008) and interaction (*P* = 0.024) but not condition effects (*P* = 0.172). The only change found with post-hoc analysis was an increase in IL-8 POST and 2 H POST vs. PRE in CON (*P* < 0.05; *g* ≥ 0.24).

MCP-1, a chemokine important for chemotaxis of monocytes, showed time (*P* = 0.009), interaction, and condition effects (both *P* ≤ 0.0001). In the REST trial, MCP-1 decreased POST and 2 H POST (both *P* < 0.05; *g* ≥ 0.65); in contrast, in the CON trial MCP-1 increased POST (*P* < 0.001*; g* = 0.83) and 2 H POST (*P* = 0.021; *g* = 0.44) and POST in the CP trial (*P* < 0.001; *g* = 0.57). Compared to the REST trial, MCP-1 was increased in the CP and CON trials POST and 2 H POST (all *P* < 0.001; *g* ≥ 0.85). There were no differences in CP and CON at any time point (*P* > 0.05).

## Discussion

The main finding of this study is that supplementation with CP (10 g/day for 8 days) before high intensity running exercise did not alter markers of GI integrity as measured by our primary outcome I-FABP. Collagen peptides also had no effect on GI permeability (L/R). However, at 2-h post-exercise, serum LPS remained low after CP supplementation (+ 14.6 pg/ml vs. REST) but significantly increased after the CON trial (+ 71.8 pg/ml), suggesting CP may have attenuated systemic increases in exercise-induced LPS.

Neither I-FABP or L/R were modulated by CP using this model of exercise-induced GI distress. As this was the first study to examine the effects of CP on permeability and GI injury in humans, direct comparisons to previous research are not possible. While there is in vitro research in Caco-2 cells showing that CP reduces NF-κB mRNA, and increases the expression of tight junction proteins that protect the intestinal barrier from insults [27], neither I-FABP or L/R were measured. In contrast to in vitro studies, we found no effect of CP on post-exercise changes in inflammatory markers (IL-6 and other cytokines) or cortisol.

Serum LPS concentrations, a measure of endotoxemia, were elevated above pre-exercise levels in the CP and CON trials at 2 h post. This is in line with some [12, 23], but not all studies, as some observed no changes in LPS in the hours post-exercise [15, 34]. A recent review suggested that LPS is typically only elevated in high ambient temperatures (e.g., ≥ 30 °C) and/or after longer duration, high intensity exercise [8]; accordingly, the high intensity of the exercise (≥ 70% $\dot{V}$O_2max_, end RPE ≥ 17; HR ≥ 180 bmp^−1^) was probably the major driver of LPS translocation in the present study. By contrast, anti-LPS antibody was unchanged at the same time point, only mildly decreasing post-exercise. As anti-LPS antibody is a marker of LPS neutralisation, it is expected to decrease when LPS increases—although this relationship isn’t always observed after exercise [12]. The lack of correlation between LPS and anti-LPS antibody could be partly explained by the limitations of the latter assay; the units of analysis are arbitrary and based on the manufacturers analysis, and not the specific population studied, and the ELISA only detects IgM antibodies. Thus, despite its use in previous exercise studies [12, 23], it may not be a valid reflection of LPS neutralisation.

Although circulatory increases in LPS are often attributed to epithelial damage and paracellular transport through the intestinal barrier, the exact mechanisms by which LPS reaches the circulation are unclear [47]. LPS may enter the circulation via endocytosis, the lymphatic system, and/or transcellular routes, which could explain why markers of intestinal integrity, permeability, and LPS, were not correlated in this present study, or in several others [15, 23, 34]. Given that LPS and I-FABP peaked at different time-points post-exercise, and L/R was collected over a longer timer period, these markers are not easy to compare. As I-FABP and LPS were measured at the same time-points, the absorption kinetics and mechanisms of their release must differ. Interestingly, LPS was more markedly elevated in the CON vs. REST trial 2 h post-exercise, but not in the CP trial. There was also a small to medium effect size for a decrease in LPS in the CP vs. CON trial (*g* = 0.48), suggesting CP may have blunted LPS efflux or served to neutralise LPS. As there were no major differences in cytokines or I-FABP and L/R in the present study, it seems unlikely that the attenuated LPS levels we observed were related to changes in inflammation and GI integrity, as suggested by the authors in animal studies [28, 29]. In addition, it might not be related to increased LPS clearance, as ALP, which detoxifies LPS [48], was unchanged—albeit, we measured this indirectly in plasma (detoxification effects are more established in the intestinal barrier [49]). Recent studies show that a high CP diet can positively influence the gut microbiota of rats [50, 51] which, in turn, may alter serum LPS levels [52], and this offers a potential explanation for the lower rise in LPS post-exercise with CP. However, this remains speculative as we did not measure gut microbiota changes in the present study and therefore future studies are needed to examine such effects. Although the mechanisms to explain why LPS was markedly higher in the CON trial—and appear attenuated in the CP trial—are not obvious from our results, the potential benefits for athletes but especially clinical populations of neutralising LPS are significant, and therefore future research should explore whether CP has a role in attenuating endotoxemia.

There were no major differences in GI symptoms between the CP and CON trials. However, given that subjective GI symptoms do not always correlate with injury and permeability markers, other non-GI factors may be involved [12, 17, 53]. Few intervention studies include markers of subjective GI symptoms as an outcome, but our findings are consistent with a similar study with the non-essential amino acid glutamine [14], which reported no benefits for GI symptoms. Interestingly, one study found that compared to water and glucose intake, whey protein, which is rich in branch-chain amino acids, significantly increased subjective GI symptoms such as bloating and nausea during 2 h of running [23]. The increased symptoms in that study could stem from the higher volume consumed compared to our study (15 g) and the fact supplements were consumed during, as opposed to before exercise. It is also possible that different amino acid compositions have disparate effects on GI symptomatology, and this warrants examination in future studies, so that exercisers are aware of which supplements may aggravate the GI system and evoke unwanted symptoms.

There are some limitations that should be acknowledged. We only standardized dietary intake in the 24 h prior to main trials and therefore cannot rule out that differences in habitual dietary intake in the days prior to this impacted the findings. In addition, while the high intensity exercise bout increased I-FABP concentrations, indicative of epithelial intestinal injury, there was no statistically significant increase in permeability after exercise, as measured by L/R—albeit there was a medium effect size for increases in lactulose compared to the REST trial. A non-statistically significant increase in L/R is not uncommon, with two similar studies in which subjects ran for 60 min at 70% $\dot{V}$O_2max_ [49] or cycled for 60 min at 70% workload max [50], also finding no significant increases in urinary L/R compared to a rest trial. We did increase the duration and exercise intensity in our study to try and exacerbate GI permeability, but the changes were perhaps too varied to reach statistical significance after adjusting for multiple comparisons. That we excluded participants with prior GI complaints is a potential limitation of the study, given that symptoms are likely more pronounced in these individuals, and therefore the supplement could be more effective.

A strength of the study is that heart rate, RPE, thermal comfort, plasma volume changes, and core temperature were not significantly different in the CON and CP trials, indicating the exercise trials were well matched for intensity, and therefore any between trials changes are unlikely due to variations in the exercise protocols. In addition, this is the first in vivo human study to examine CP in this context and provides a rationale for future studies. Future research is warranted with longer dosing strategies, higher doses of CP, different timings of intake, other acute GI stress models, and/or in volunteers with marked GI symptoms or chronic exercise-induced gastrointestinal conditions. Future studies should also include additional markers associated with GI distress and inflammatory bowel diseases such as ZO-1, alpha-1-antitrypsin and calprotectin.

## Conclusion

This study demonstrates that supplementation with CP does not alter exercise-induced changes in GI injury, permeability, and inflammation, or modify commonly reported GI symptoms. Although CP had no effect on our primary outcome measure I-FABP, a novel and intriguing finding was the greater LPS levels in the CON vs. non-exercise rest trial. While LPS was a not our main outcome measure, and we saw no statistically significant differences between the CON and CP trial, future research exploring a role for CP in modulating endotoxemia is warranted.

## Data Availability

Not available.
